# Dynamic changes in plasma Epstein–Barr virus DNA load during treatment have prognostic value in nasopharyngeal carcinoma: a retrospective study

**DOI:** 10.1002/cam4.1381

**Published:** 2018-03-01

**Authors:** Sha‐Sha He, Yan Wang, Yong Bao, Xiu‐Yu Cai, Xing‐Li Yang, Dan‐Ming Chen, Yong Chen, Li‐Xia Lu

**Affiliations:** ^1^ State Key Laboratory of Oncology in South China Collaborative Innovation Center for Cancer Medicine Sun Yat sen University Cancer Center Guangzhou Guangdong China; ^2^ Department of Radiation Oncology Sun Yat‐Sen University Cancer Center Guangzhou Guangdong China; ^3^ Department of Radiation Oncology The first affiliated hospital of Sun Yat‐Sen University Guangzhou Guangdong China; ^4^ Department of VIP Region Sun Yat‐Sen University Cancer Center Guangzhou Guangdong China

**Keywords:** Epstein–Barr virus DNA, nasopharyngeal carcinoma, survival, treatment

## Abstract

Circulating plasma Epstein–Barr virus DNA (EBV DNA) is related to tumor recurrence and metastasis and has potential as a dynamic, sensitive, and specific marker in nasopharyngeal carcinoma (NPC). We investigated the clinical significance of assessing plasma EBV DNA load at various time points during treatment. Patients with NPC (*n *=* *949) for whom plasma EBV DNA load was measured by real‐time quantitative polymerase chain reaction (RT‐qPCR) before treatment (pre‐EBV) and at midtreatment (mid‐EBV), end of treatment (end‐EBV), and 3 months after completing treatment (3 m‐EBV) were retrospectively assessed. Receiver operating characteristic (ROC) curve analysis was used to identify the optimal EBV DNA cutoff point for each time point. Overall survival (OS), distant metastasis‐free survival (DMFS), and progression‐free survival (PFS) were compared using Kaplan–Meier estimates. High pre‐EBV, high mid‐EBV, high end‐EBV, and high 3 m‐EBV were all associated with significantly poorer OS, DMFS, and PFS in the entire cohort. Detectable end‐EBV and 3 m‐EBV was associated with significantly poorer OS, DMFS, and PFS. Among patients with detectable end‐EBV, adjuvant therapy significantly improved OS (HR 2.419; 95% CI 1.297–4.51, *P *=* *0.03) and DMFS (HR 2.45; 95% CI 1.243–4.828, *P *=* *0.04), but not PFS (*P *=* *0.17). EBV DNA represents a dynamic biomarker for monitoring treatment and predicting survival in NPC. Assessing plasma EBV DNA before, during, and after chemoradiotherapy could be clinically valuable and enable selection of patients most likely to benefit from additional therapy and improve assessment of treatment response and disease surveillance. Further multicenter prospective investigations are warranted.

## Introduction

Nasopharyngeal carcinoma (NPC) has a unique epidemiology that includes regional, racial, dialectal, and familial aggregation. Southern China has the highest incidence of NPC in China, peaking at 29 per 100,000 person‐years 1. In addition to established genetic and environmental factors, plasma Epstein–Barr viral DNA (EBV DNA) load is a well‐recognized biomarker in NPC. EBV DNA is used for diagnosis, screening and even as a predictor of clinical outcome and correlates positively with disease stage 2, 3 as well as tumor burden. The gross tumor volume of the cervical lymph nodes (GTVnd, *P *<* *0.001) and total tumor volume (GTVtotal, *P *<* *0.001) are both closely related to pretreatment EBV DNA. Combination of EBV DNA with tumor volume has been reported to improve prognostication and clinical decision making 4. Moreover, EBV DNA is assessed as a component of surveillance for local recurrence and metastasis in NPC 5, 6. Patients with recurrent and metastatic NPC have significantly higher EBV DNA than patients in clinical remission (*P *<* *0.01). Therefore, EBV DNA could represent a sensitive tumor marker for monitoring tumor recurrence and metastasis after radiotherapy in NPC 7.

Most studies have analyzed the value of pretreatment or post‐treatment EBV DNA. However, as EBV DNA is a dynamic biomarker, assessment of EBV DNA during therapy and follow‐up may provide more accurate prognostication. A study in advanced‐stage NPC revealed detectable EBV DNA after completion of 4 weeks of radiotherapy was associated with poorer clinical outcome in terms of distant failure, progression‐free survival, and overall survival 8. Thus, in this study, we retrospectively assessed real‐time quantitative PCR EBV DNA data at several time points during therapy for patients with NPC and compared several clinical outcomes (OS, DMFS, PFS). Moreover, subgroup analyses based on dynamic changes in EBV DNA were conducted to generate risk stratification and potentially enable individualized therapy. Furthermore, we explored the impact of adjuvant therapy on patients with detectable end‐EBV.

## Materials and Methods

### Patients

Data were obtained from the medical records of 949 patients with NPC treated between January 2010 and October 2013 at Sun Yat‐sen University Cancer Center (SYSUCC). Demographic data (including age, sex, smoking, and treatment) were obtained from the hospital electronic record system. All patients were restaged according to the 8th American Joint Committee on Cancer/Union for International Cancer Control (AJCC/UICC) staging system 9. Staging work‐up included direct fiber‐optic nasopharyngoscopy, nasopharyngeal and neck magnetic resonance imaging (MRI), abdominal ultrasound, chest radiography, entire body bone scan and/or positron emission tomography‐computed tomography (PET‐CT), and plasma EBV DNA copy number.

This study was approved by the Research Ethics Committee of Sun Yat‐sen University Cancer Center, Guangzhou, China. The inclusion criteria for this study were as follows: (1) newly pathologically diagnosed primary undifferentiated nonkeratinized carcinoma, (2) no history of previous anticancer therapy and first received radiotherapy with or without chemotherapy at our hospital, and (3) adequate clinical information in the medical records, especially EBV DNA copy number at each time point.

### Treatment

The therapeutic protocol was strictly based on the guidelines of SYSUCC, which have been described previously 7. All patients received intensity‐modulated radiation therapy (IMRT) with or without chemotherapy: IMRT alone for stage I; IMRT with concurrent chemotherapy for stage II; concurrent chemoradiotherapy with or without neoadjuvant or adjuvant chemotherapy for stage III and IVA‐B. IMRT was 2.12–2.24 Gy fractions daily for 5 days/week, up to a total of 68–72 Gy to the primary tumor, 62–66 Gy to involved areas of the neck, and 50 Gy to uninvolved areas. Neoadjuvant or adjuvant chemotherapy consisted of cisplatin with 5‐fluorouracil or cisplatin with taxanes, or all three together, administered triweekly for 2 or 3 cycles. Concurrent chemotherapy consisted of cisplatin administered triweekly or weekly until the end of radiotherapy. Cetuximab and nimotuzumab were also given as an adjuvant targeted therapy on 48 and 34 patients, respectively.

### Quantification of plasma EBV DNA

Measurement of plasma EBV DNA was performed at baseline (pre‐EBV) and at the midpoint of radiotherapy (mid‐EBV), end of therapy (end‐EBV), and 3 months after therapy (3 m‐EBV) using real‐time quantitative PCR. The assay was developed for detection of plasma EBV DNA and targets the BamHI‐W fragment region of the EBV genome 10 and was performed using 2× TaqMan reagent (Roche), the amplification primers W‐44F (50‐AGTCTCTGCCTCAGGGCA‐30) and W‐119R (50‐ACAGAGGGCCTGTCCACCG‐30), and the dual‐labeled fluorescent probe W‐67T (50‐[FAM]‐CACTGTCTGTAAAGTCCAGCCTCC‐[TAMRA]‐30). At our institution, a plasma EBV DNA concentration of <10^3^ copies/mL was defined as undetectable. The cutoff values for each time point were defined using ROC curve analyses: The cutoff levels chosen to define low and high EBV DNA were pre‐EBV, 2500 copies/mL; mid‐EBV, 871 copies/mL; end‐EBV, 721 copies/mL; and 3 m‐EBV, 211 copies/mL.

### Follow‐up

The primary endpoint for this study was overall survival (OS), measured from the date of first NPC diagnosis to either the date of death or loss to follow‐up. The secondary endpoints were distant metastasis‐free survival (DMFS, to distant relapse or patient censorship at last follow‐up) and progression‐free survival (PFS, to locoregional failure, distant failure, or death from any cause, whichever occurred first). All patients were routinely followed up after therapy: every 3 months during the first 2 years, every 6 months during years 3–5, and annually thereafter. The surveillance work‐up included normal routine assessments, as well as EBV DNA.

### Statistical analysis

Statistical analysis was performed using IBM SPSS software version 22.0 (IBM Corp., Armonk, NY, USA). Survival outcomes were estimated using the Kaplan–Meier method and compared with the log‐rank test. All analyses were two‐sided; the level of significance was set at *P *<* *0.05.

## Results

A total of 949 patients treated between January 2010 and October 2013 were retrospectively enrolled. Median follow‐up period in our study was 55.43 months (range, 2.67–88.65 months). Median age was 45 years (range, 11–77 years), and 79.7% of patients were male. All patients had nonkeratinizing undifferentiated squamous cell carcinoma. Overall, 438 (46.2%) patients had pretreatment EBV DNA over 2500 copies/mL. The baseline clinical features of the patients are shown in Table 1.

**Table 1:** Summary of the clinicopathological features of the 949 patients with nasopharyngeal carcinoma

Characteristic	Total (No, %)
	949 (100)
Age (years)	
<45	522 (55.0)
≥45	427 (45.0)
Sex	
Male	756 (79.7)
Female	193 (20.3)
Smoking	
Yes	212 (28.3)
No	737 (71.7)
Overall stage (8th edition)	
I	91 (9.6)
II	246 (25.9)
III	450 (47.4)
IVAB	162 (17.1)
T category	
T1	222 (23.4)
T2	153 (16.1)
T3	447 (47.1)
T4	127 (13.4)
N category	
N0	180 (19.0)
N1	614 (64.7)
N2	111 (11.7)
N3	44 (4.6)
Treatment	
IMRT	238 (25.1)
CT + IMRT	711 (74.9)
EBV DNA (copies/mL)	
<2500	511 (53.8)
≥2500	438 (46.2)

T, tumor; N, node; NPC, nasopharyngeal carcinoma; IMRT, intensity‐modulated radiation therapy; CT, chemotherapy.

### Survival outcomes according to the 8th edition of the AJCC

**Figure 1:**
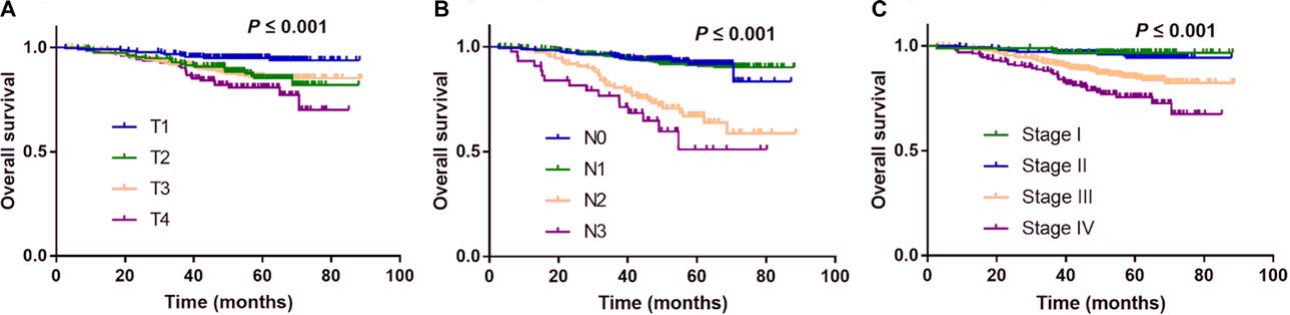
Effect of AJCC classification and stage on overall survival. Kaplan–Meier overall survival curves for all 154 patients with NPC stratified by T category (A), N category (B), and overall stage (C).

We restaged the patients according to the 8th AJCC staging system and compared the survival outcomes for each category and stage. Advanced T category, N category, and overall stage were associated with poorer survival (all *P *≤* *0.001; Fig. 1).

### Survival outcomes according to EBV DNA at various time points

**Figure 2:**
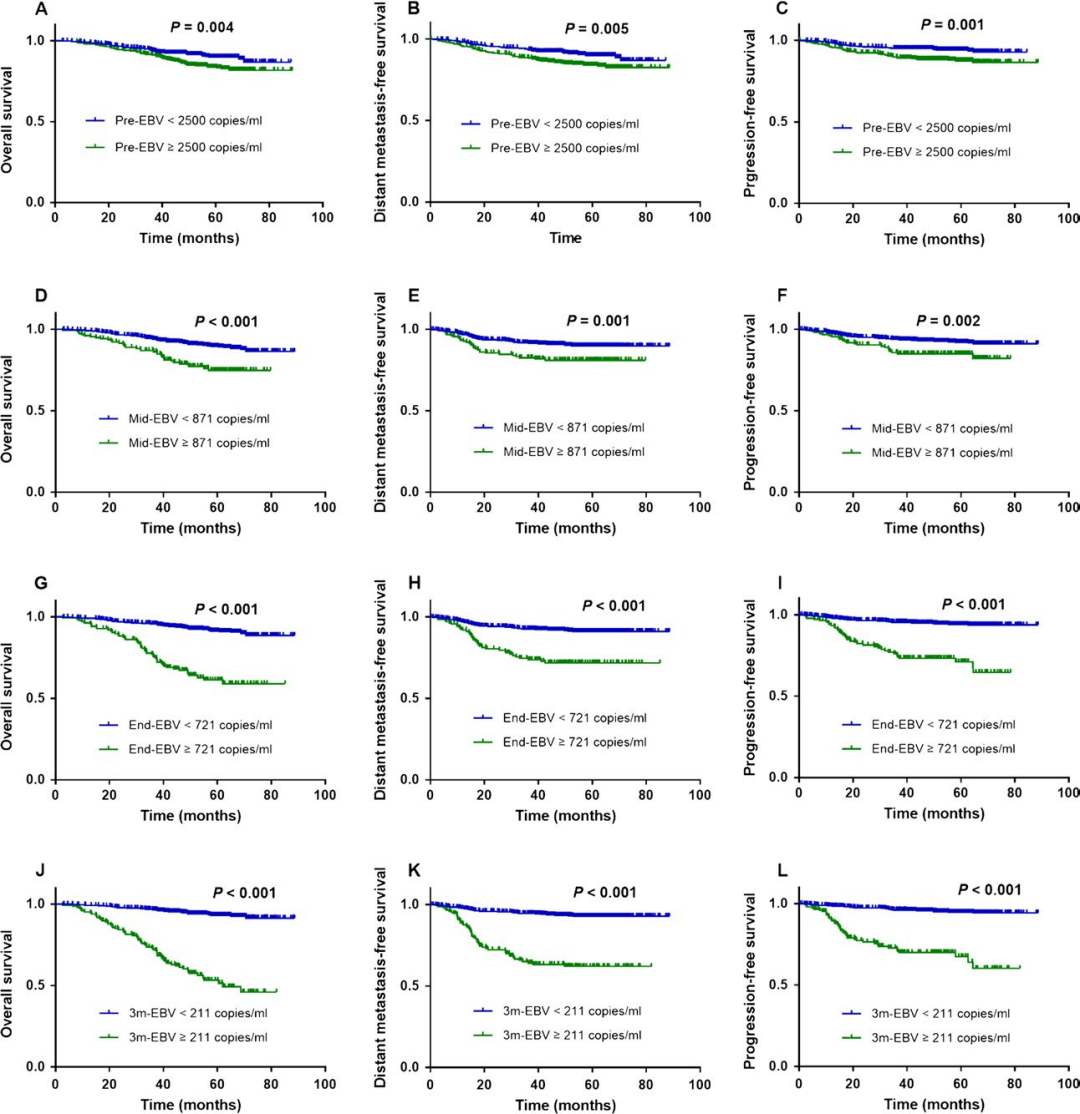
Effect of plasma EBV DNA at different treatment time points on survival outcomes. Kaplan–Meier overall survival, distant metastasis‐free survival, and progression‐free survival curves for all 154 patients with NPC stratified by pre‐EBV (<2500 vs. ≥2500 copies/mL; A, B, C), mid‐EBV (<871 vs. ≥871 copies/mL; D, E, F), end‐EBV (<721 vs. ≥721 copies/mL; G, H, I), and 3 m‐EBV (<211 vs. ≥211 copies/mL; J, K, L).

Four‐year OS, DMFS, and PFS for the entire cohort were 89.6%, 88.9%, and 91.7%, respectively. Using the optimal EBV DNA cutoff value identified for each time point of treatment, patients with high EBV DNA had poorer survival rates. Patients with <2500 pre‐EBV copies/mL achieved better OS (*P *=* *0.004; Fig. 2A), DMFS (*P *=* *0.005; Fig. 2B), and PFS (*P *=* *0.001; Fig. 2C) than patients with higher pre‐EBV. Patients with <871 mid‐EBV copies/mL obtained better OS (*P *<* *0.001; Fig. 1D), DMFS (*P *=* *0.001; Fig. 2E), and PFS (*P *=* *0.002; Fig. 2F) than patients with higher mid‐EBV. Patients with <721 end‐EBV copies/mL had higher OS (*P *<* *0.001; Fig. 2G), DMFS (*P *<* *0.001; Fig. 2H), and PFS (*P *<* *0.001; Fig. 2I) than patients with higher end‐EBV. Patients with <211 3 m‐EBV copies/mL had better OS (*P *<* *0.001; Fig. 2J), DMFS (*P *<* *0.001; Fig. 2K), and PFS (*P *<* *0.001; Fig. 2L) than patients with higher 3 m‐EBV.

**Figure 3:**
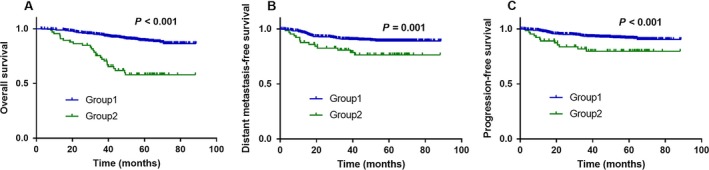
Detectable end‐EBV and detectable 3 m‐EBV are associated with poor survival outcomes. Kaplan–Meier overall survival (A), distant metastasis‐free survival (B), and progression‐free survival (C) curves for all 154 patients with NPC stratified as Group 1 (undetectable end‐EBV and undetectable 3 m‐EBV; detectable end‐EBV and undetectable 3 m‐EBV; undetectable end‐EBV and detectable 3 m‐EBV) and Group 2 (detectable end‐EBV and detectable 3 m‐EBV).

Next, we classified the patients into Group 1 (undetectable end‐EBV and undetectable 3 m‐EBV; detectable end‐EBV and undetectable 3 m‐EBV; undetectable end‐EBV and detectable 3 m‐EBV) or Group 2 (detectable end‐EBV and detectable 3 m‐EBV). Patients in Group 2 (detectable end‐EBV and 3 m‐EBV) had significantly poorer OS [hazard ratio (HR) 4.71; 95% CI 2.159–10.27, *P *<* *0.001; Fig. 3A), DMFS (HR 2.396, 95% CI 1.073–5.351, *P *=* *0.001; Fig. 3B), and PFS (HR 2.71, 95% CI 1.085–6.766, *P *<* *0.001; Fig. 3C) than the patients in Group 1.

### Subgroup analysis of patients with detectable end‐EBV

**Figure 4:**
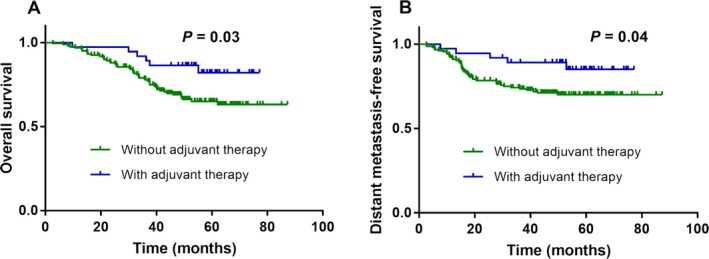
Effect of adjuvant therapy on survival outcomes in patients with detectable end‐EBV. Kaplan–Meier overall survival (A) and distant metastasis‐free survival (B) curves for the 196 patients with detectable end‐EBV stratified by with or without adjuvant therapy.

There were 137 of 949 patients who expressed detectable end‐EBV in our study, the median age was 45 years old, and the median EBV copy number was 9500 copies/mL. In this subgroup, the median OS rate was 47.5 months. Finally, we assessed the prognostic value of end‐EBV for guiding individualized therapy. Patients with detectable end‐EBV who received adjuvant therapy (chemotherapy and/or targeted therapy) achieved better OS (HR 2.419; 95% CI 1.297–4.51, *P *=* *0.03; Fig. 4A) and DMFS (HR 2.45; 95% CI 1.243–4.828, *P *=* *0.04; Fig. 4B), but not PFS (*P *=* *0.17), compared to patients with detectable end‐EBV who did not receive adjuvant therapy.

## Discussion

Analysis of EBV DNA has proven useful for early detection, staging, prognostication, and monitoring of treatment response in NPC. The clinical value of pretreatment EBV DNA has been widely accepted. There was a cohort of 90 patients with early stage who received radiation therapy only; patients with distant failure had remarkably higher pretreatment EBV DNA levels than those without failure (a median of 13,219 copies/mL vs. a median of 423 copies/mL) 11. Due to the fact that the patients with Stage I‐II disease addressed infrequent failure, patients with both advanced‐stage and high EBV DNA levels had actually high distant failure rate. Thus, there was a hypothesis that high levels of pre‐EBV in early‐stage patients may express with a similar probability of distant failure compared to patients with advanced‐stage disease, as reported about 37% vs. 20–40% 12, 13. In our study, high levels of pre‐EBV with early stage (I+II) demonstrated higher OS than low levels of pre‐EBV with advanced stage (III+IVAB) (*P *=* *0.032), but not DMFS (*P *=* *0.172).

However, as previous studies have mainly assessed EBV DNA at a single time point, usually pretreatment, little is known about the clinical value of dynamic changes in EBV DNA during treatment. The value of dynamic changes in EBV DNA during and after treatment remains controversial. Previous studies indicated a reduction in EBV DNA to 0 copies/mL after treatment was associated with significantly better tumor control and patient survival. Jin‐Ching Lin et al. found patients with persistently detectable plasma EBV DNA had significantly worse overall survival (*P *<* *0.001) and relapse‐free survival (*P *<* *0.001) than patients with undetectable EBV DNA 1 week after the completion of radiotherapy 14. A meta‐analysis of 14 prospective and retrospective comparative studies conducted by Zhang et al. 15 concluded patients with more rapid EBV DNA clearance rates (i.e., shorter half‐life) achieved significantly better OS than patients with slower EBV DNA clearance rates and indicated pre‐DNA, mid‐DNA, and post‐DNA, and the EBV DNA clearance rate were all prognostic factors for OS, DMFS, LRFS, and PFS. Plasma EBV DNA level reflects tumor burden, and elimination of EBV DNA from the circulation after surgery was very fast. Another research studied the kinetics of circulating EBV DNA in the plasma of NPC patients, they revealed there was an initial rise in plasma EBV DNA concentration during the first week of radiation therapy, which was consistent with the liberation of EBV DNA after therapy‐induced cancer cell death. And it began to decay with a median half‐life of 3.8 days 16. The linearity of the plot of the logarithm of EBV DNA concentration against time supported a first‐order kinetics model of EBV DNA elimination which was also consistent with the clearance 17.

Patients with advanced‐stage NPC revealed detectable mid‐EBV DNA was associated with significantly poorer distant failure (HR 12.02; 95% CI 2.78–51.93, *P *=* *0.0009), PFS (HR 4.05, 95% CI 1.89–8.67, *P *=* *0.0003), and OS (HR 3.29, 95% CI 1.37–7.89, *P *=* *0.0077) 18. A large‐scale cohort study combined EBV DNA and total tumor volume (GTV total) and found EBV DNA >0 copies/mL and GTV total ≥ 30 cm^3^ were strongly associated with poorer prognosis in patients treated with IMRT 4. However, there was a finding that patients with a small tumor and high EBV DNA had a poorer prognosis than patients with a large tumor and low EBV DNA, while patients with low EBV DNA had a favorable prognosis, regardless of tumor volume 19.

Considering the reveal of patients with NPC having high EBV DNA levels are at greater risk of developing distant metastases 20, which is currently the major cause of failure of treatment for NPC, there are some studies aim at excavating the gene‐related therapies, which is the developing cancer therapeutic method. Latent membrane protein 1 (LMP1), an EBV‐encoded oncogene, is expressed in 65% of NPC tumor biopsies 21. One study suggested the EBV DNA sequence shares homology with LMP1 oncogene deletions, which are associated with aggressive behavior in NPC 22. In addition, LMP1 was described as target antigen for therapeutic augmentation of cytotoxic T‐lymphocyte (CTL) responses in patients with NPC 23. Thus, in the future, an EBV‐specific CTL vaccine may represent an immunotherapeutic strategy that could potentially reduce recurrence or distant metastasis in NPC.

### Study limitations

This study has some limitations. First, it was designed as a retrospective study and may lead to the potential bias; Second, the population enrolled in the study was still small. Moreover, the effects of radiotherapy may have been differently influenced by the chemotherapeutic combination used. Despite these limitations, this study highlights the importance of EBV DNA detection and its value for predicting survival through its dynamic changes.

## Conclusions

Patients with high EBV DNA before, during, and after therapy have a poorer prognosis than patients with low or undetectable EBV DNA at the same time points. Furthermore, we found adjuvant therapy improved the survival outcomes of patients with detectable end‐EBV DNA. Therefore, EBV DNA may be useful for identifying high‐risk patients and guiding individual therapy in NPC; it is reasonable to suggest that patients with detectable EBV DNA after basic therapy may require closer surveillance and may benefit from adjuvant therapy.

## Conflict of Interest

The authors declare that they have no competing interests.

## Supporting information


**Data S1**. The authenticity of this article has been validated by uploading the key raw data onto the Research Data Deposit public platform (http://www.researchdata.org.cn), and the RDD number is RDDA2018000487.
