# Supplementary material for: Dynamic changes in plasma Epstein–Barr virus DNA load during treatment have prognostic value in nasopharyngeal carcinoma: a retrospective study
Source: Cancer Med. 2018 Mar 1;7(4):1110–7. doi: 10.1002/cam4.1381 (PMC5911595; doi:10.1002/cam4.1381)
Supplement: Supplementary file 1 — Data S1. The authenticity of this article has been validated by uploading the key raw data onto the Research Data Deposit public platform (http://www.researchdata.org.cn), and the RDD number is RDDA2018000487. [file CAM4-7-1110-s001.docx]

**Data S1**. The authenticity of this article has been validated by uploading the key raw data onto the Research Data Deposit public platform (www.researchdata.org.cn), the approval is progress and the RDD number will be provided before accepted.
